# Attributable burden in patients with carbapenem-nonsusceptible gram-negative respiratory infections

**DOI:** 10.1371/journal.pone.0229393

**Published:** 2020-02-21

**Authors:** Ying P. Tabak, Anita Sung, Gang Ye, Latha Vankeepuram, Vikas Gupta, Eilish McCann

**Affiliations:** 1 Digital Health, Medical Affairs, Becton, Dickinson and Company, Franklin Lakes, New Jersey, United States of America; 2 Center for Observational and Real-World Evidence (CORE), Merck & Co., Inc., Kenilworth, New Jersey, United States of America; University of South Florida, UNITED STATES

## Abstract

**Objective:**

We aimed to describe the clinical and economic burden attributable to carbapenem-nonsusceptible (C-NS) respiratory infections.

**Methods:**

This retrospective matched cohort study assessed clinical and economic outcomes of adult patients (aged ≥18 years) who were admitted to one of 78 acute care hospitals in the United States with nonduplicate C-NS and carbapenem-susceptible (C-S) isolates from a respiratory source. A subset analysis of patients with principal diagnosis codes denoting bacterial pneumonia or other diagnoses was also conducted. Isolates were classified as community- or hospital-onset based on collection time. A generalized linear mixed model method was used to estimate the attributable burden for mortality, 30-day readmission, length of stay (LOS), cost, and net gain/loss (payment minus cost) using propensity score-matched C-NS versus C-S cohorts.

**Results:**

For C-NS cases, mortality (25.7%), LOS (29.4 days), and costs ($81,574) were highest in the other principal diagnosis, hospital-onset subgroup; readmissions (19.4%) and net loss (-$9522) were greatest in the bacterial pneumonia, hospital-onset subgroup. Mortality and readmissions were not significantly higher for C-NS cases in any propensity score-matched subgroup. Significant C-NS–attributable burden was found for both other principal diagnosis subgroups for LOS (hospital-onset: 3.7 days, *P* = 0.006; community-onset: 1.5 days, *P*<0.001) and cost (hospital-onset: $12,777, *P*<0.01; community-onset: $2681, *P*<0.001).

**Conclusions:**

Increased LOS and cost burden were observed in propensity score-matched patients with C-NS compared with C-S respiratory infections; the C-NS–attributable burden was significant only for patients with other principal diagnoses.

## Introduction

Respiratory infections, such as bacterial pneumonia, rank among the leading causes of mortality in the United States (U.S.) and are responsible for more than 900,000 hospitalizations each year [1,2]. While the proportion of hospitalizations related to pneumonia appears to be declining, a study found that hospitalization rates for bacterial pneumonia caused by 2 gram-negative pathogens (*Klebsiella* and *Pseudomonas spp*.) increased significantly from 2002 through 2011 (by 35% and 23%, respectively; *P*<0.001) [3,4]. In addition, gram-negative pathogens are commonly implicated in hospital-acquired bacterial pneumonia [5,6].

Guideline-recommended antibiotics for the empiric treatment of gram-negative respiratory infections include antipseudomonal penicillins, cephalosporins, carbapenems, monobactams, fluoroquinolones, aminoglycosides, and polymyxins [7,8]. Despite the array of antibiotic classes available for the treatment of these infections, antibiotic resistance is an ongoing and increasing global health problem that reduces treatment options [9]. Antibiotic resistance is estimated to result in 23,000 deaths each year in the U.S., with an estimated additional cost to the healthcare system of $20 billion annually [10]. Several antimicrobial surveillance programs of respiratory isolates have documented decreased susceptibility to a variety of antibiotics, including the carbapenem class [5,11,12].

Carbapenem-nonsusceptible (C-NS) isolates from any infection source are associated with poor patient outcomes, including fewer resolved infections after 1 month, increased hospital length of stay (LOS), and increased risk of mortality [13–16]. In this study we examined the clinical and economic impact specific to respiratory infections due to culture-confirmed gram-negative C-NS pathogens. In particular, we compared the burden of C-NS infections with that of carbapenem-susceptible (C-S) infections in a retrospective analysis of microbiological and administrative data from 78 U.S. hospitals.

## Materials and methods

### Data source

For this retrospective matched cohort study of adult patients aged ≥18 years who were admitted to one of 78 U.S. acute care hospitals with respiratory source isolates that were C-NS or C-S, we used the BD Insights Research Database (Becton, Dickinson and Company, Franklin Lakes, NJ, USA) to obtain electronically captured patient-level microbiological and administrative data [17–19]. The dataset was collected from January 1, 2013, to September 30, 2015, prior to the transition from the International Classification of Diseases, Ninth Revision, Clinical Modification (ICD-9-CM) to the 10th version in the U.S., and included microbiological data (specimen collection time, source, and culture and susceptibility results), hospital data, and post-discharge administrative data (principal diagnosis, discharge disposition, payer, hospital LOS, hospital cost, and payment received by the hospital). The study dataset was a deidentified and limited retrospective dataset exempted from patient consent by the New England Institutional Review Board (Wellesley, MA, USA).

### Study population

Nonduplicate (the first isolate of any species obtained from a patient per 30-day period) respiratory isolates from adult in-patients that were tested for carbapenem susceptibility were included in the study. Patients were stratified by their principal ICD-9-CM diagnosis code and a subset of patients had an ICD-9-CM code that denoted bacterial pneumonia (S1 Table).

### Definitions

#### Carbapenem-nonsusceptible versus carbapenem-susceptible cases

Gram-negative respiratory isolates were classified as C-NS if they were determined to be “resistant” or “intermediate” to imipenem or meropenem for *Pseudomonas aeruginosa* or *Acinetobacter baumannii* (as ertapenem is intrinsically not active against these pathogens), or to imipenem, meropenem, or ertapenem for *Enterobacteriaceae* (new taxonomy: Enterobacterales): *Escherichia coli*, *Klebsiella pneumoniae*, *Proteus mirabilis*, *Enterobacter cloacae*, *Enterobacter aerogenes*, *Serratia marcescens*, *Citrobacter freundii*, *Morganella morganii*. Procedures and systems used for susceptibility testing and microbiological reporting could be variable across institutions. The definitions of “resistant” or “intermediate” were determined using the interpretative results for each hospital report in the laboratory information system.

#### Infection-onset period

Specimen collection time (<3 versus ≥3 days from hospital admission) dictated the classification of isolates as either community-onset or hospital-onset, respectively.

### Statistical analysis

#### Propensity score matching

Four subgroups sorted by 1) principal diagnosis (bacterial pneumonia versus other) and 2) infection-onset period (community-onset versus hospital-onset) were created: Group 1, bacterial pneumonia, community-onset; Group 2, bacterial pneumonia, hospital-onset; Group 3, other principal diagnosis, community-onset; Group 4, other principal diagnosis, hospital-onset. Using C-NS and C-S as a binary variable, a propensity score model was developed for each of the 4 subgroups, thereby permitting adjustment of potential confounders. The propensity models included the following potential confounding factors: age, sex, payer, intensive care unit admission status, mechanical ventilation status, principal diagnosis-based disease categories (using the Clinical Classification Software [CCS] categories [for other principal diagnosis subgroups only]) [20], type of gram-negative organism(s), exposure risk (number of days from admission to onset of infection [for hospital-onset subgroups only]), number of hospital admissions in the 90 days before the index admission, and hospital attributes (teaching status, hospital size, geographic location). We also included an Acute Laboratory Risk of Mortality Score (ALaRMS) as an aggregate measure of clinical severity [21]. The ALaRMS uses patient demographics and 23 numeric laboratory test results to score the probability of in-hospital mortality. Laboratory parameters assessed include serum chemistry (albumin, aspartate transaminase, alkaline phosphatase, blood urea nitrogen, calcium, creatinine, glucose, potassium, sodium, and total bilirubin), hematology and coagulation parameters (bands, hemoglobin, partial thromboplastin time, prothrombin time, international normalized ratio, platelets, and white blood cell count), arterial blood gas (partial pressure of carbon dioxide, partial pressure of oxygen, and pH value), and cardiac markers (brain natriuretic peptide, creatinine phosphokinase-MB, pro-brain natriuretic peptide, and troponin I or troponin T).

Based on the propensity score generated using the aforementioned parameters, C-NS cases were matched with C-S cases by applying the Greedy 1-to-1 nearest-neighbor matching algorithm within each subgroup [22]. The matching caliper used for each propensity score matching program was 0.25. For both the matched C-NS and C-S cases, outcomes were compared within each subgroup to ensure comparability.

#### Outcomes

In-hospital mortality, 30-day readmission, LOS, cost, and net gain/loss, defined as total payment received minus total cost, were the outcomes of interest for this study.

#### Estimating attributable clinical and economic burden

C-NS–attributable burden for each outcome was estimated using the generalized linear mixed model (GLMM) method on the propensity score-matched C-NS and C-S cases within each subgroup. Inter-hospital variations and skewed distributions were accounted for by the GLMM approach. Specifically, we used the random intercept logistic regression models (a special form of GLMM) for the 2 categorical outcomes of in-hospital mortality and 30-day readmission. For the continuous outcomes—LOS, cost, and net gain/loss—we used the regular GLMM with the gamma distribution as the transformation function and “hospital” as the random effect. *P* values <0.05 were viewed as statistically significant. All analyses were conducted using the Statistical Analysis System (SAS) version 9.4 (SAS Institute, Cary, NC, USA).

## Results

### Patient characteristics

**Fig 1:**
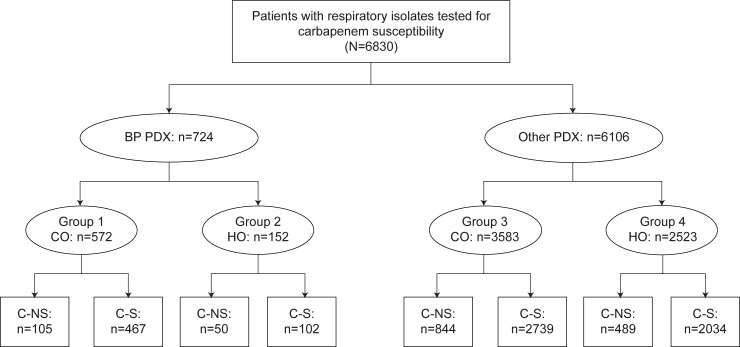
C-NS versus C-S case tree. BP, bacterial pneumonia; C-NS, carbapenem nonsusceptible; C-S, carbapenem susceptible; CO, community-onset; HO, hospital-onset; PDX, principal diagnosis.

Carbapenem susceptibility testing results were available for 6830 admissions with gram-negative isolates from a respiratory source. Of those, 724 had principal diagnosis codes denoting bacterial pneumonia (Group 1, 572 community-onset; Group 2, 152 hospital-onset) and 6106 had another principal diagnosis (Group 3, 3583 community-onset; Group 4, 2523 hospital-onset) (Fig 1).

Baseline characteristics for each subgroup, sorted by propensity score matching for C-NS versus C-S, are shown in Table 1 (S2 Table shows characteristics before and after matching). After matching, each subgroup contained equal numbers of C-NS and C-S cases (Group 1, 101; Group 2, 42; Group 3, 742; Group 4, 463). Within each of the 4 subgroups, the potential confounding factors were largely balanced. In the overall matched cohort, most patients were ≥55 years of age, and *P*. *aeruginosa* was the pathogen type observed most frequently (57.5%-82.2%), followed by “other gram-negative” (5.9%-27.4%) and polymicrobial infections (0%-15.1%). Most patients had an ALaRMS score in the first, second, or third quartile (59.5%-81.9%). Higher proportions of patients with an ALaRMS score in the fourth quartile (denoting the highest in-hospital mortality risk) were observed in the hospital-onset subgroups (27.6% and 40.5% for Groups 2 and 4, respectively) versus community-onset subgroups (18.1% and 21.8% for Groups 1 and 3, respectively). In the other principal diagnosis subgroups (Groups 3 and 4), the most frequent CCS disease categories were diseases of the respiratory system (eg, respiratory failure, chronic obstructive pulmonary disease, emphysema), infections/parasitic diseases (eg, septicemia and infections of other organs or systems), injury, and poisoning.

**Table 1:** Distribution of patient characteristics after propensity score matching.

Variables, n (%)	Group 1: BP PDX, CO		Group 2: BP PDX, HO		Group 3: Other PDX, CO		Group 4: Other PDX, HO	
	C-NS (n = 101)	C-S (n = 101)	C-NS (n = 42)	C-S (n = 42)	C-NS (n = 742)	C-S (n = 742)	C-NS (n = 463)	C-S (n = 463)
**Sex**								
Female	52 (51.5)	51 (50.5)	24 (57.1)	21 (50.0)	328 (44.2)	301 (40.6)	162 (35.0)	168 (36.3)
Male	49 (48.5)	50 (49.5)	18 (42.9)	21 (50.0)	414 (55.8)	441 (59.4)	301 (65.0)	295 (63.7)
**Age group, years**								
18–34	17 (16.8)	21 (20.8)	3 (7.1)	5 (11.9)	238 (32.1)	256 (34.5)	48 (10.4)	56 (12.1)
35–44	9 (8.9)	7 (6.9)	1 (2.4)	1 (2.4)	83 (11.2)	76 (10.2)	35 (7.6)	33 (7.1)
45–54	9 (8.9)	8 (7.9)	2 (4.8)	2 (4.8)	80 (10.8)	73 (9.8)	70 (15.1)	60 (13.0)
55–64	15 (14.9)	12 (11.9)	14 (33.3)	11 (26.2)	101 (13.6)	101 (13.6)	107 (23.1)	92 (19.9)
65–74	30 (29.7)	36 (35.6)	14 (33.3)	7 (16.7)	128 (17.3)	125 (16.8)	123 (26.6)	138 (29.8)
75–84	13 (12.9)	13 (12.9)	5 (11.9)	11 (26.2)	83 (11.2)	82 (11.1)	57 (12.3)	61 (13.2)
85 or older	8 (7.9)	4 (4.0)	3 (7.1)	5 (11.9)	29 (3.9)	29 (3.9)	23 (5.0)	23 (5.0)
**Payer**								
Medicare	51 (50.5)	51 (50.5)	24 (57.1)	23 (54.8)	370 (49.9)	380 (51.2)	234 (50.5)	240 (51.8)
Medicaid	6 (5.9)	9 (8.9)	5 (11.9)	2 (4.8)	51 (6.9)	58 (7.8)	39 (8.4)	37 (8.0)
Private/other	44 (43.6)	41 (40.6)	13 (31.0)	17 (40.5)	321 (43.3)	304 (41.0)	190 (41.0)	186 (40.2)
**ALaRMS Score**^**a**^								
1st quartile	36 (35.6)	41 (40.6)	15 (35.7)	11 (26.2)	307 (41.4)	312 (42.0)	115 (24.8)	128 (27.6)
2nd quartile	25 (24.8)	23 (22.8)	4 (9.5)	7 (16.7)	153 (20.6)	139 (18.7)	98 (21.2)	103 (22.2)
3rd quartile	18 (17.8)	16 (15.8)	6 (14.3)	9 (21.4)	148 (19.9)	148 (19.9)	112 (24.2)	104 (22.5)
4th quartile	22 (21.8)	21 (20.8)	17 (40.5)	15 (35.7)	134 (18.1)	143 (19.3)	138 (29.8)	128 (27.6)
**Number of hospital admissions in the 90 days before index admission**								
0	59 (58.4)	59 (58.4)	26 (61.9)	27 (64.3)	405 (54.6)	402 (54.2)	323 (69.8)	313 (67.6)
1	26 (25.7)	25 (24.8)	11 (26.2)	12 (28.6)	232 (31.3)	228 (30.7)	85 (18.4)	96 (20.7)
>1	16 (15.8)	17 (16.8)	5 (11.9)	3 (7.1)	105 (14.2)	112 (15.1)	55 (11.9)	54 (11.7)
**Intensive care unit admission status**								
No	69 (68.3)	69 (68.3)	31 (73.8)	32 (76.2)	528 (71.2)	526 (70.9)	183 (39.5)	181 (39.1)
Yes	32 (31.7)	32 (31.7)	11 (26.2)	10 (23.8)	214 (28.8)	216 (29.1)	280 (60.5)	282 (60.9)
**Mechanical ventilation status**								
No	70 (69.3)	74 (73.3)	29 (69.0)	30 (71.4)	501 (67.5)	489 (65.9)	239 (51.6)	238 (51.4)
Yes	31 (30.7)	27 (26.7)	13 (31.0)	12 (28.6)	241 (32.5)	253 (34.1)	224 (48.4)	225 (48.6)
**Exposure risk (# of days from admission to onset of infection)**								
1st quartile	N/A	N/A	22 (54.2)	12 (28.6)	N/A	N/A	92 (19.9)	83 (17.9)
2nd quartile	N/A	N/A	3 (7.1)	6 (14.3)	N/A	N/A	94 (20.3)	99 (21.4)
3rd quartile	N/A	N/A	6 (14.3)	12 (28.6)	N/A	N/A	99 (21.4)	112 (24.2)
4th quartile	N/A	N/A	11 (26.2)	12 (28.6)	N/A	N/A	178 (38.4)	169 (36.5)
**Type of gram-negative organism**								
*Pseudomonas aeruginosa*	79 (78.2)	83 (82.2)	34 (81.0)	34 (81.0)	562 (75.7)	564 (76.0)	269 (58.1)	266 (57.5)
Polymicrobial	14 (13.9)	12 (11.9)	0 (0)	4 (9.5)	98 (13.2)	93 (12.5)	69 (14.9)	70 (15.1)
Other gram-negative	8 (7.9)	6 (5.9)	8 (19.0)	4 (9.5)	82 (11.1)	85 (11.5)	125 (27.0)	127 (27.4)
**PDX-based CCS disease category**^**b**^								
Diseases of the respiratory system	N/A	N/A	N/A	N/A	163 (22.0)	184 (24.8)	89 (19.2)	94 (20.3)
Endocrine, nutritional, and metabolic diseases and immunity disorders	N/A	N/A	N/A	N/A	219 (29.5)	215 (29.0)	29 (6.3)	27 (5.8)
Infectious and parasitic diseases	N/A	N/A	N/A	N/A	192 (25.9)	192 (25.9)	102 (22.0)	93 (20.1)
Injury and poisoning	N/A	N/A	N/A	N/A	62 (8.4)	59 (8.0)	91 (19.7)	94 (20.3)
Diseases of the circulatory system	N/A	N/A	N/A	N/A	28 (3.8)	22 (3.0)	49 (10.6)	54 (11.7)
Diseases of the digestive system	N/A	N/A	N/A	N/A	16 (2.2)	12 (1.6)	31 (6.7)	32 (6.9)
Neoplasms	N/A	N/A	N/A	N/A	8 (1.1)	10 (1.3)	18 (3.9)	17 (3.7)
Missing PDX	N/A	N/A	N/A	N/A	20 (2.7)	16 (2.2)	16 (3.5)	12 (2.6)
All other CCS	N/A	N/A	N/A	N/A	34 (4.6)	32 (4.3)	38 (8.2)	40 (8.6)
**Hospital teaching status**								
Nonteaching	60 (59.4)	61 (60.4)	34 (81.0)	34 (81.0)	354 (47.7)	365 (49.2)	264 (57.0)	269 (58.1)
Teaching	41 (40.6)	40 (39.6)	8 (19.0)	8 (19.0)	388 (52.3)	377 (50.8)	199 (43.0)	194 (41.9)
**Hospital size (number of beds)**								
≤300	34 (33.7)	25 (24.8)	18 (42.9)	15 (35.7)	144 (19.4)	133 (17.9)	75 (16.2)	64 (13.8)
>300	67 (66.3)	76 (75.2)	24 (57.1)	27 (64.3)	598 (80.6)	609 (82.1)	388 (83.8)	399 (86.2)
**Geographic location (regions**)								
Midwest	17 (16.8)	13 (12.9)	6 (14.3)	1 (2.4)	101 (13.6)	84 (11.3)	92 (19.9)	82 (17.7)
Northeast	1 (1.0)	0 (0)	2 (4.8)	2 (4.8)	30 (4.0)	29 (3.9)	17 (3.7)	18 (3.9)
South	72 (71.3)	78 (77.2)	30 (71.4)	37 (88.1)	562 (75.7)	589 (79.4)	326 (70.4)	342 (73.9)
West	11 (10.9)	10 (9.9)	4 (9.5)	2 (4.8)	49 (6.6)	40 (5.4)	28 (6.0)	21 (4.5)

All post-matching variables were well-balanced with all *P* values >0.05, indicating no significant differences between C-NS and C-S cohorts within each of the 4 groups (see S2 Table for pre- versus post-matching details). ALaRMS, Acute Laboratory Risk of Mortality Score; BP, bacterial pneumonia; C-NS, carbapenem nonsusceptible; C-S, carbapenem susceptible; CCS, Clinical Classification Software; CO, community-onset; HO, hospital-onset; N/A, not applicable; PDX, principal diagnosis.

^a^ALaRMS quartile cutoff was based on the distribution within each of the 4 subgroups prior to propensity score matching.

^b^As determined by CCS software.

### Propensity score-matched results

**Fig 2:**
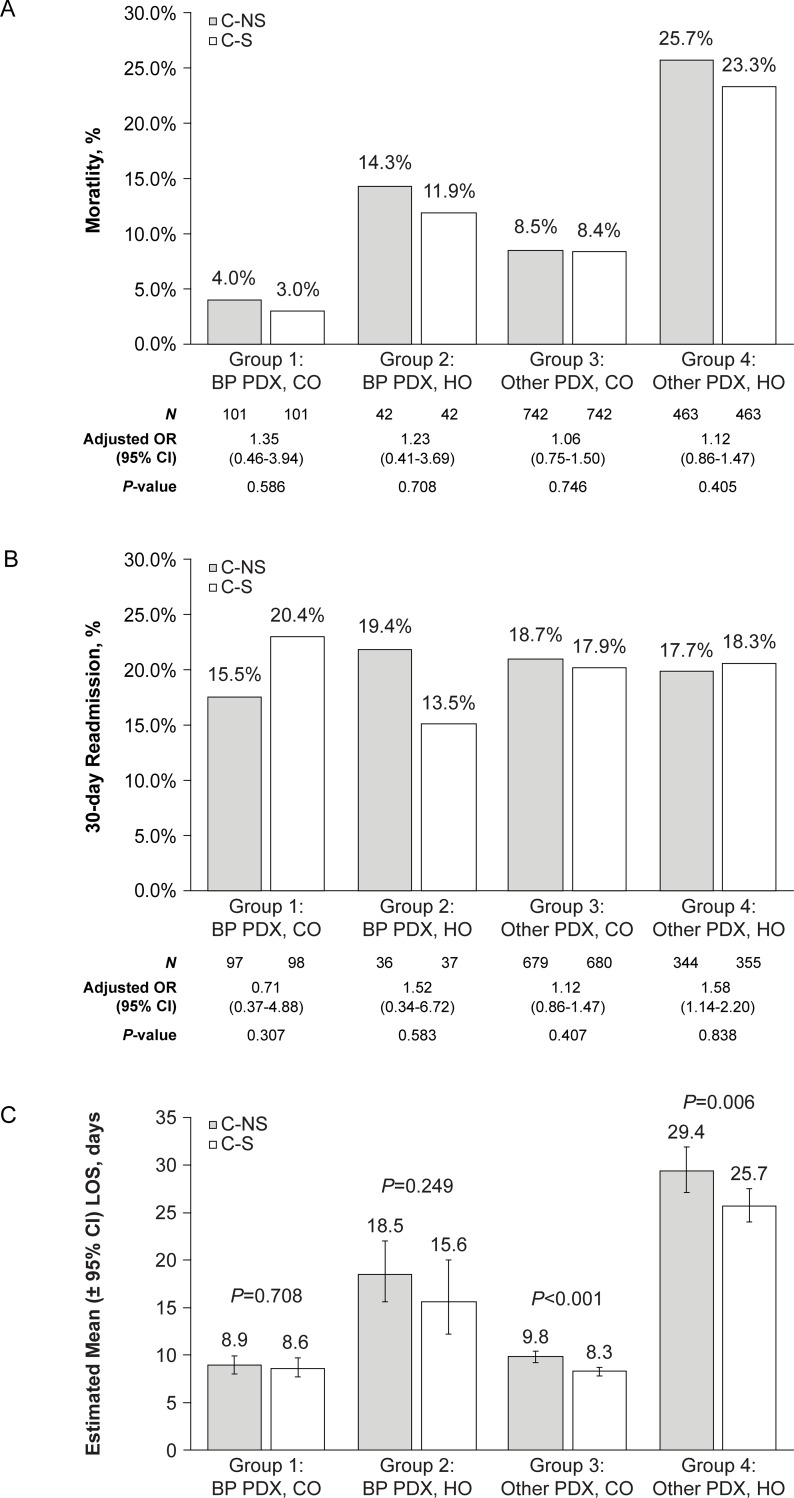
Outcomes by propensity score-matched patient cohorts (carbapenem-nonsusceptible [C-NS; grey bars] cases versus carbapenem-susceptible [C-S; open bars] cases). (A) Mortality. (B) 30-Day readmission. 30-Day readmission was only measured in patients who were alive at discharge. (C) Length of stay (LOS). BP, bacterial pneumonia; CI, confidence interval; CO, community onset; HO, hospital onset; PDX, principal diagnosis.

In-hospital mortality varied from 4% to 25.7% for C-NS infections and from 3% to 23.3% for C-S infections across the 4 subgroups (Fig 2A). The mortality rate was highest in C-NS cases in Group 4 (other principal diagnosis, hospital-onset). Higher mortality was found for hospital-onset subgroups compared with community-onset subgroups. In each subgroup, mortality was higher for C-NS infections than C-S infections (adjusted odds ratios indicated a 6% to 35% increase in risk of mortality), but the differences between C-NS and C-S infections were not statistically significant.

Readmissions occurred in 15.5% to 19.4% of patients with C-NS infections (Fig 2B); the range was 13.5% to 20.4% for C-S infections, with the greatest readmission rate observed in Group 1 (bacterial pneumonia, community-onset). The difference between C-NS and C-S cases was not significant in any of the subgroups. There were no consistent relationships between carbapenem susceptibility status, infection-onset period, or principal diagnosis and the likelihood of readmission.

Mean LOS was 8.9 days to 29.4 days for C-NS cases and 8.3 days to 25.7 days for C-S cases, with the longest LOS observed in Group 4 (29.4 days; 95% confidence interval [CI]: 27.1, 31.9) (Fig 2C). In all subgroups the mean LOS was greater for C-NS versus C-S cases, and this difference was statistically significant for Groups 3 and 4 (+1.5 days; *P*<0.001 and +3.7 days; *P* = 0.006, respectively). Mean LOS for hospital-onset cases was either double or triple that of community-onset cases (principal diagnosis of bacterial pneumonia or other, respectively).

**Fig 3:**
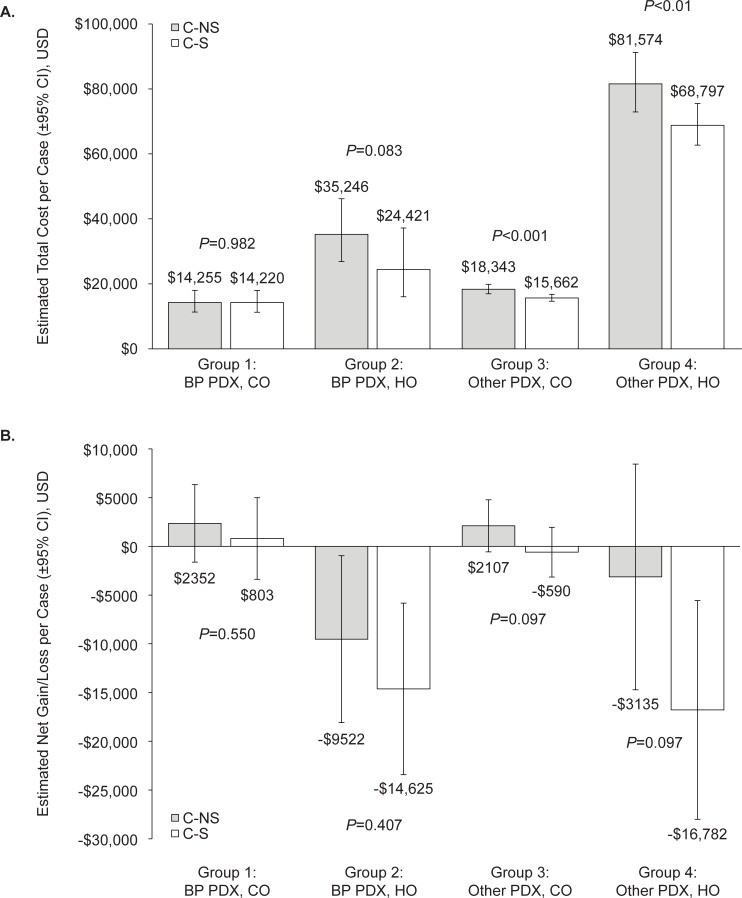
Total cost (A) and net gain/loss (B) by propensity score-matched patient cohorts. BP, bacterial pneumonia; C-NS, carbapenem nonsusceptible; C-S, carbapenem susceptible; CI, confidence interval; CO, community-onset; HO, hospital-onset; PDX, principal diagnosis; USD, United States dollars.

The mean total cost incurred by hospitals for each C-NS infection ranged from $14,255 to $81,574, and from $14,220 to $68,797 for each C-S infection (Fig 3A). Group 4 (other principal diagnosis, hospital-onset) accounted for the highest figure in both of these ranges, with the highest overall cost observed for C-NS infections ($81,574; 95% CI: $72,928, $91,245). In all 4 subgroups the cost of C-NS infections was greater than that of C-S infections; this difference was statistically significant for Groups 3 and 4 (+$2681; *P*<0.001 and +$12,777; *P*<0.01, respectively).

The financial impact to the hospital was limited in Groups 1 and 3, with either a net gain or a small loss per case (Fig 3B). Larger losses were observed in Groups 2 and 4, with the largest negative balance associated with C-S infections in Group 4 (-$16,782; 95% CI: -$28,000, -$5,563). This suggests that the payments received for hospital-onset infections are not sufficient to cover their cost, leaving the hospital with a negative balance. In all subgroups, the net gain or loss was worse with C-S infections than C-NS infections, although the differences between C-S and C-NS cases were not statistically significant.

## Discussion

The burden attributable to respiratory infections has been well-described in the literature; however, few studies have focused specifically on the impact of gram-negative respiratory C-NS infections in comparison with C-S infections, and there are no studies of which we are aware that have evaluated both clinical and economic outcomes and the resulting financial impact to the hospital.

The in-hospital mortality rates reported here are broadly comparable to others in the literature, with previously reported rates ranging from 14.5% to 60.6% for C-NS isolates [14–16,23,24]. The highest mortality rate in this study (25.7%) was in patients with other conditions (a principal diagnosis other than bacterial pneumonia) who developed a concomitant respiratory infection during their hospital stay. This analysis also showed that the risk of death was numerically higher for C-NS versus C-S infections (although not statistically significantly higher), indicating that a concomitant respiratory infection that is C-NS may be more serious for patients than C-S infections. Future studies with larger sample sizes would be needed to further examine the mortality burden. For 30-day readmission, rates were generally high (up to 20.4%); however, no clear trends relating to C-NS status, principal diagnosis, or onset of infection were evident from this analysis.

Both LOS and cost ranged widely across the 4 subgroups; however, LOS is likely a key component of the overall cost. Our findings for gram-negative infections fall within the expected range of costs from the literature (the total cost of hospital-acquired respiratory infections of any kind is estimated to range from $22,300 to $99,598) [25–27]. In all subgroups, LOS and costs for C-NS infections were higher than for C-S infections, with a statistically significant C-NS–attributable burden observed in both other principal diagnosis subgroups. As with mortality, the highest burden for LOS and cost was borne by patients with other principal diagnoses who developed a respiratory infection once admitted to the hospital. These patients therefore represent an important target for anyone wishing to effectively manage the burden associated with gram-negative respiratory infections.

Regarding the balance of costs and payment received, we showed that hospitals lose money for each patient with a hospital-onset infection, but lose a smaller amount for C-NS infections compared with C-S infections (these differences were not statistically significant). Given the greater complexity of C-NS infections and the reduced number of appropriate treatment options, this result is perhaps surprising. Investigating and understanding the nature of payer reimbursement to hospitals for C-NS infections may provide some insight.

The strengths of this study include the large number of patients with positive respiratory isolate cultures from diverse hospital settings (eg, small versus large, teaching versus nonteaching). In addition, the propensity score matching method enabled us to balance potential confounding factors for the outcomes and minimize possible sources of bias. Nevertheless, propensity score matching has its own limitations, which could potentially affect outcomes. Approximately 10% of unmatched C-NS cases were dropped from further analysis. Identification of a subset of patients with ICD-9-CM codes indicative of bacterial pneumonia was intended to reduce the potential for including patients who are colonized with gram-negative organisms but who do not have a clinically meaningful infection. However, we only identified a relatively low number of patients with a principal diagnosis of bacterial pneumonia and a C-NS isolate (n = 155). The lack of secondary diagnoses in the dataset limited further confirmation of diagnosis codes. Thus, we were not able to rule out the possibility that positive culture results were due to colonization. Nevertheless, given the worse outcomes observed even for those with other diagnoses as the primary reason for hospital admission, there is a good likelihood that these patients had a true infection. Locally applied microbiological assessments and interpretation may vary from one institution to the next, which can be considered a limitation to the study. The use of ICD-9-CM codes may also be a limitation to this study, in that codes are primarily used for billing purposes and may be over-used, under-used or misused, and therefore do not accurately capture the patient’s clinical situation. Chart review and radiographic imaging confirmation, in conjunction with ICD-9-CM codes and culture confirmation, would more accurately and fully characterize each case. In addition, analysis of antibiotic utilization before and during the index hospitalization may help to identify patients with clinically relevant infections and provide insights into any relationship between antibiotic prescribing practices and observed clinical and economic outcomes. Further analyses that identify the key drivers of outcomes in this patient population, such as patient characteristics or the relationship between payer type and payments received, would also be of great interest.

In conclusion, C-NS–attributable burden was observed in propensity score-matched patients with culture-confirmed gram-negative respiratory infections for mortality (risk of death numerically increased by up to 35%), LOS (significantly extended by up to 3.7 days), and cost (significant additional costs in excess of $12,000). This C-NS–attributable burden was greatest in patients admitted to hospital with other underlying conditions who then developed a hospital-onset respiratory infection. These data illustrate how carbapenem nonsusceptibility can increase the burden of respiratory infections, especially in patients hospitalized with potentially complex and serious clinical conditions.

## Supporting information

S1 Table Principal diagnosis codes (ICD-9-CM). CMS, Centers for Medicare and Medicaid Services; ICD-9-CM, International Classification of Diseases, Ninth Revision, Clinical Modification; NOS, not otherwise specified; VAP, ventilator-associated pneumonia.
(DOCX)

S2 Table Pre- and post-matching of baseline characteristics: Respiratory groups 1, 2, 3, and 4. Respiratory group 2 had the fewest patients so variable groupings were modified from respiratory groups 1, 3, and 4 in order to conduct statistical tests. ALaRMS, Acute Laboratory Risk of Mortality Score; C-NS, carbapenem nonsusceptible; C-S, carbapenem susceptible; CCS, Clinical Classification Software; ICU, intensive care unit; PDX, principal diagnosis.
(DOCX)
