# Supplementary material for: Attributable burden in patients with carbapenem-nonsusceptible gram-negative respiratory infections
Source: PLoS One. 2020 Feb 21;15(2):e0229393. doi: 10.1371/journal.pone.0229393 (PMC7034906; doi:10.1371/journal.pone.0229393)
Supplement: S1 Table — CMS, Centers for Medicare and Medicaid Services; ICD-9-CM, International Classification of Diseases, Ninth Revision, Clinical Modification; NOS, not otherwise specified; VAP, ventilator-associated pneumonia. (DOCX) [file pone.0229393.s001.docx]

**Supporting Table S1.** Principal diagnosis codes (ICD-9-CM)

| **Principal diagnosis** | **Diagnosis code (CMS)** | **Short description** |
| --- | --- | --- |
| 482 | 4820 | *K. pneumoniae* pneumonia |
| 482.1 | 4821 | Pseudomonal pneumonia |
| 482.83 | 48283 | Pneumonia (other gram-negative bacteria) |
| 482.9 | 4829 | Bacterial pneumonia NOS |
| 483.8 | 4838 | Pneumonia (other special organism) |
| 485 | 485 | Bronchopneumonia (organism NOS) |
| 486 | 486 | Pneumonia (organism NOS) |
| 997.31 | 99731 | VAP |

CMS, Centers for Medicare and Medicaid Services; ICD-9-CM, International Classification of Diseases, Ninth Revision, Clinical Modification; NOS, not otherwise specified; VAP, ventilator-associated pneumonia.
