# Supplementary material for: Attributable burden in patients with carbapenem-nonsusceptible gram-negative respiratory infections
Source: PLoS One. 2020 Feb 21;15(2):e0229393. doi: 10.1371/journal.pone.0229393 (PMC7034906; doi:10.1371/journal.pone.0229393)
Supplement: S2 Table — Respiratory group 2 had the fewest patients so variable groupings were modified from respiratory groups 1, 3, and 4 in order to conduct statistical tests. ALaRMS, Acute Laboratory Risk of Mortality Score; C-NS, carbapenem nonsusceptible; C-S, carbapenem susceptible; CCS, Clinical Classification Software; ICU, intensive care unit; PDX, principal diagnosis. (DOCX) [file pone.0229393.s002.docx]

**Supporting Table S2a.** Pre- and post-matching of baseline characteristics: Respiratory group 1 with bacterial pneumonia primary diagnosis and community onset

| **Variables** | **Pre-matching** | | | | | **Post-matching (1:1 match)** | | | | |
| --- | --- | --- | --- | --- | --- | --- | --- | --- | --- | --- |
|  | **C-S (n=467)** | | **C-NS (n=105)** | |  | **C-S (n=101)** | | **C-NS (n=101)** | |  |
|  | n | Column % | n | Column % | ***P* value** | n | Column % | n | Column % | ***P* value** |
| **Sex** |  |  |  |  | **0.246** |  |  |  |  | **0.888** |
| Female | 211 | 45.2 | 54 | 51.4 |  | 51 | 50.5 | 52 | 51.5 |  |
| Male | 256 | 54.8 | 51 | 48.6 |  | 50 | 49.5 | 49 | 48.5 |  |
| **Age group, years** |  |  |  |  | **<0.001** |  |  |  |  | **0.825** |
| 18-34 | 40 | 8.6 | 17 | 16.2 |  | 21 | 20.8 | 17 | 16.8 |  |
| 35-44 | 12 | 2.6 | 10 | 9.5 |  | 7 | 6.9 | 9 | 8.9 |  |
| 45-54 | 26 | 5.6 | 11 | 10.5 |  | 8 | 7.9 | 9 | 8.9 |  |
| 55-64 | 92 | 19.7 | 15 | 14.3 |  | 12 | 11.9 | 15 | 14.9 |  |
| 65-74 | 136 | 29.1 | 31 | 29.5 |  | 36 | 35.6 | 30 | 29.7 |  |
| 75-84 | 103 | 22.1 | 13 | 12.4 |  | 13 | 12.9 | 13 | 12.9 |  |
| 85 and over | 58 | 12.4 | 8 | 7.6 |  | 4 | 4.0 | 8 | 7.9 |  |
| **Payer** |  |  |  |  | **0.005** |  |  |  |  | **0.703** |
| Medicare | 314 | 67.2 | 53 | 50.5 |  | 51 | 50.5 | 51 | 50.5 |  |
| Medicaid | 23 | 4.9 | 7 | 6.7 |  | 9 | 8.9 | 6 | 5.9 |  |
| Private/other | 130 | 27.8 | 45 | 42.9 |  | 41 | 40.6 | 44 | 43.6 |  |
| ALaRMS Score |  |  |  |  | **0.193** |  |  |  |  | **0.908** |
| 1st quartile | 121 | 25.9 | 37 | 35.2 |  | 41 | 40.6 | 36 | 35.6 |  |
| 2nd quartile | 119 | 25.5 | 27 | 25.7 |  | 23 | 22.8 | 25 | 24.8 |  |
| 3rd quartile | 112 | 24.0 | 18 | 17.1 |  | 16 | 15.8 | 18 | 17.8 |  |
| 4th quartile | 115 | 24.6 | 23 | 21.9 |  | 21 | 20.8 | 22 | 21.8 |  |
| **Number of hospital admissions in the 90 days before index admission** | | | | | **<0.001** |  |  |  |  | **0.975** |
| 0 | 346 | 74.1 | 59 | 56.2 |  | 59 | 58.4 | 59 | 58.4 |  |
| 1 | 91 | 19.5 | 26 | 24.8 |  | 25 | 24.8 | 26 | 25.7 |  |
| >1 | 30 | 6.4 | 20 | 19.0 |  | 17 | 16.8 | 16 | 15.8 |  |
| **ICU admission status** |  |  |  |  | **<0.001** |  |  |  |  | **1.000** |
| No | 397 | 85.0 | 71 | 67.6 |  | 69 | 68.3 | 69 | 68.3 |  |
| Yes | 70 | 15.0 | 34 | 32.4 |  | 32 | 31.7 | 32 | 31.7 |  |
| **Mechanical ventilation status** | | | | | **<0.001** |  |  |  |  | **0.534** |
| No | 411 | 88.0 | 72 | 68.6 |  | 74 | 73.3 | 70 | 69.3 |  |
| Yes | 56 | 12.0 | 33 | 31.4 |  | 27 | 26.7 | 31 | 30.7 |  |
| **Type of gram-negative organism** | | | | | **<0.001** |  |  |  |  | **0.776** |
| *Pseudomonas aeruginosa* | 264 | 56.5 | 81 | 77.1 |  | 83 | 82.2 | 79 | 78.2 |  |
| Polymicrobial | 32 | 6.9 | 16 | 15.2 |  | 12 | 11.9 | 14 | 13.9 |  |
| Other gram-negative | 171 | 36.6 | 8 | 7.6 |  | 6 | 5.9 | 8 | 7.9 |  |
| **Hospital teaching status** | | | | | **<0.001** |  |  |  |  | **0.886** |
| Nonteaching | 385 | 82.4 | 62 | 59.0 |  | 61 | 60.4 | 60 | 59.4 |  |
| Teaching | 82 | 17.6 | 43 | 41.0 |  | 40 | 39.6 | 41 | 40.6 |  |
| **Hospital size (number of beds)** | | | | | **0.005** |  |  |  |  | **0.164** |
| ≤300 | 226 | 48.4 | 35 | 33.3 |  | 25 | 24.8 | 34 | 33.7 |  |
| >300 | 241 | 51.6 | 70 | 66.7 |  | 76 | 75.2 | 67 | 66.3 |  |
| **Geographic location (regions)** | | | | | **0.010** |  |  |  |  | **0.648** |
| Midwest | 42 | 9.0 | 19 | 18.1 |  | 13 | 12.9 | 17 | 16.8 |  |
| Northeast | 7 | 1.5 | 1 | 1.0 |  | . | . | 1 | 1.0 |  |
| South | 391 | 83.7 | 74 | 70.5 |  | 78 | 77.2 | 72 | 71.3 |  |
| West | 27 | 5.8 | 11 | 10.5 |  | 10 | 9.9 | 11 | 10.9 |  |

ALaRMS, Acute Laboratory Risk of Mortality Score; C-NS, carbapenem nonsusceptible; C-S, carbapenem susceptible; ICU, intensive care unit.

**Supporting Table S2b.** Pre- and post-matching of baseline characteristics: Respiratory group 2 with bacterial pneumonia primary diagnosis and hospital onset

| **Variables** | **Pre-matching** | | | | | **Post-matching (1:1 match)** | | | | |
| --- | --- | --- | --- | --- | --- | --- | --- | --- | --- | --- |
|  | **C-S (n=102)** | | **C-NS (n=50)** | |  | **C-S (n=42)** | | **C-NS (n=42)** | |  |
|  | n | Column % | n | Column % | ***P* value** | n | Column % | n | Column % | ***P* value** |
| **Sex** | | | | | **0.639** |  |  |  |  | **0.512** |
| Female | 53 | 52.0 | 28 | 56.0 |  | 21 | 50.0 | 24 | 57.1 |  |
| Male | 49 | 48.0 | 22 | 44.0 |  | 21 | 50.0 | 18 | 42.9 |  |
| **Age ≥65 years** | | | | | **0.019** |  |  |  |  | **0.827** |
| No | 31 | 30.4 | 25 | 50.0 |  | 19 | 45.2 | 20 | 47.6 |  |
| Yes | 71 | 69.6 | 25 | 50.0 |  | 23 | 54.8 | 22 | 52.4 |  |
| **Medicare** | | | | | **0.009** |  |  |  |  | **0.826** |
| No | 29 | 28.4 | 25 | 50.0 |  | 19 | 45.2 | 18 | 42.9 |  |
| Yes | 73 | 71.6 | 25 | 50.0 |  | 23 | 54.8 | 24 | 57.1 |  |
| **Exposure risk (# of days from admission to onset of infection)** | | | | | **0.187** |  |  |  |  | **0.112** |
| 1st quartile | 51 | 50.0 | 16 | 32.0 |  | 22 | 52.4 | 12 | 28.6 |  |
| 2nd quartile | 11 | 10.8 | 6 | 12.0 |  | 3 | 7.1 | 6 | 14.3 |  |
| 3rd quartile | 18 | 17.6 | 14 | 28.0 |  | 6 | 14.3 | 12 | 28.6 |  |
| 4th quartile | 22 | 21.6 | 14 | 28.0 |  | 11 | 26.2 | 12 | 28.6 |  |
| **ALaRMS score was ≥53 (median)** | | | | | **0.567** |  |  |  |  | **0.827** |
| No | 54 | 52.9 | 24 | 48.0 |  | 20 | 47.6 | 21 | 50.0 |  |
| Yes | 48 | 47.1 | 26 | 52.0 |  | 22 | 52.4 | 21 | 50.0 |  |
| **Number of hospital admissions in the 90 days before index admission** | | | | | **0.493** |  |  |  |  | **0.821** |
| 0 | 67 | 65.7 | 30 | 60.0 |  | 27 | 64.3 | 26 | 61.9 |  |
| ≥1 | 35 | 34.3 | 20 | 40.0 |  | 15 | 35.7 | 16 | 38.1 |  |
| **Mechanical ventilation status** | | | | | **0.005** |  |  |  |  | **0.811** |
| No | 83 | 81.4 | 30 | 60.0 |  | 30 | 71.4 | 29 | 69.0 |  |
| Yes | 19 | 18.6 | 20 | 40.0 |  | 12 | 28.6 | 13 | 31.0 |  |
| ***Pseudomonas aeruginosa*** | | | | | **0.003** |  |  |  |  | **1.000** |
| No | 43 | 42.2 | 9 | 18.0 |  | 8 | 19.0 | 8 | 19.0 |  |
| Yes | 59 | 57.8 | 41 | 82.0 |  | 34 | 81.0 | 34 | 81.0 |  |
| **Hospital size (number of beds)** | | | | | **0.795** |  |  |  |  | **0.503** |
| ≤300 | 41 | 40.2 | 19 | 38.0 |  | 15 | 35.7 | 18 | 42.9 |  |
| >300 | 61 | 59.8 | 31 | 62.0 |  | 27 | 64.3 | 24 | 57.1 |  |

Respiratory group 2 had the fewest patients so variable groupings were modified from respiratory groups 1, 3, and 4 in order to conduct statistical tests. ALaRMS, Acute Laboratory Risk of Mortality Score; C-NS, carbapenem nonsusceptible; C-S, carbapenem susceptible.

**Supporting Table S2c.** Pre- and post-matching of baseline characteristics: Respiratory group 3 without bacterial pneumonia primary diagnosis and community onset

| **Variables** | **Pre-matching** | | | | | **Post-matching (1:1 match)** | | | | |
| --- | --- | --- | --- | --- | --- | --- | --- | --- | --- | --- |
|  | **CS (n=2739)** | | **CNS (n=844)** | |  | **CS (n=742)** | | **CNS (n=742)** | |  |
|  | n | Column % | n | Column % | ***P* value** | n | Column % | n | Column % | ***P* value** |
| **Sex** | | | | | **0.001** |  |  |  |  | **0.150** |
| Female | 1111 | 40.6 | 396 | 46.9 |  | 301 | 40.6 | 328 | 44.2 |  |
| Male | 1628 | 59.4 | 448 | 53.1 |  | 441 | 59.4 | 414 | 55.8 |  |
| **Age group, years** | | | | | **<0.001** |  |  |  |  | **0.970** |
| 18-34 | 377 | 13.8 | 299 | 35.4 |  | 256 | 34.5 | 238 | 32.1 |  |
| 35-44 | 138 | 5.0 | 109 | 12.9 |  | 76 | 10.2 | 83 | 11.2 |  |
| 45-54 | 267 | 9.7 | 86 | 10.2 |  | 73 | 9.8 | 80 | 10.8 |  |
| 55-64 | 522 | 19.1 | 102 | 12.1 |  | 101 | 13.6 | 101 | 13.6 |  |
| 65-74 | 674 | 24.6 | 133 | 15.8 |  | 125 | 16.8 | 128 | 17.3 |  |
| 75-84 | 524 | 19.1 | 86 | 10.2 |  | 82 | 11.1 | 83 | 11.2 |  |
| 85 and over | 237 | 8.7 | 29 | 3.4 |  | 29 | 3.9 | 29 | 3.9 |  |
| **Payer** | | | | | **<0.001** |  |  |  |  | **0.593** |
| Medicare | 1600 | 58.4 | 407 | 48.2 |  | 380 | 51.2 | 370 | 49.9 |  |
| Medicaid | 130 | 4.7 | 80 | 9.5 |  | 58 | 7.8 | 51 | 6.9 |  |
| Private/other | 1009 | 36.8 | 357 | 42.3 |  | 304 | 41.0 | 321 | 43.3 |  |
| **ALaRMS score** | | | | | **<0.001** |  |  |  |  | **0.800** |
| 1st quartile | 577 | 21.1 | 361 | 42.8 |  | 312 | 42.0 | 307 | 41.4 |  |
| 2nd quartile | 703 | 25.7 | 178 | 21.1 |  | 139 | 18.7 | 153 | 20.6 |  |
| 3rd quartile | 757 | 27.6 | 165 | 19.5 |  | 148 | 19.9 | 148 | 19.9 |  |
| 4th quartile | 702 | 25.6 | 140 | 16.6 |  | 143 | 19.3 | 134 | 18.1 |  |
| **Number of hospital admissions in the 90 days before index admission** | | | | | **<0.001** |  |  |  |  | **0.873** |
| 0 | 1922 | 70.2 | 411 | 48.7 |  | 402 | 54.2 | 405 | 54.6 |  |
| 1 | 561 | 20.5 | 277 | 32.8 |  | 228 | 30.7 | 232 | 31.3 |  |
| >1 | 256 | 9.3 | 156 | 18.5 |  | 112 | 15.1 | 105 | 14.2 |  |
| **ICU admission status** | | | | | **<0.001** |  |  |  |  | **0.909** |
| No | 1688 | 61.6 | 618 | 73.2 |  | 526 | 70.9 | 528 | 71.2 |  |
| Yes | 1051 | 38.4 | 226 | 26.8 |  | 216 | 29.1 | 214 | 28.8 |  |
| **Mechanical ventilation status** | | | | | **0.635** |  |  |  |  | **0.509** |
| No | 1829 | 66.8 | 571 | 67.7 |  | 489 | 65.9 | 501 | 67.5 |  |
| Yes | 910 | 33.2 | 273 | 32.3 |  | 253 | 34.1 | 241 | 32.5 |  |
| **Type of gram-negative organism** | | | | | **<0.001** |  |  |  |  | **0.910** |
| *Pseudomonas aeruginosa* | 1209 | 44.1 | 653 | 77.4 |  | 564 | 76.0 | 562 | 75.7 |  |
| Polymicrobial | 239 | 8.7 | 109 | 12.9 |  | 93 | 12.5 | 98 | 13.2 |  |
| Other gram-negative | 1291 | 47.1 | 82 | 9.7 |  | 85 | 11.5 | 82 | 11.1 |  |
| **PDX-based CCS disease category^a^** | | | | | **<0.001** |  |  |  |  | **0.907** |
| Missing PDX | 116 | 4.2 | 21 | 2.5 |  | 16 | 2.2 | 20 | 2.7 |  |
| Injury and poisoning | 186 | 6.8 | 79 | 9.4 |  | 59 | 8.0 | 62 | 8.4 |  |
| Diseases of the circulatory system | 307 | 11.2 | 28 | 3.3 |  | 22 | 3.0 | 28 | 3.8 |  |
| Diseases of the digestive system | 87 | 3.2 | 17 | 2.0 |  | 12 | 1.6 | 16 | 2.2 |  |
| Diseases of the respiratory system | 916 | 33.4 | 170 | 20.1 |  | 184 | 24.8 | 163 | 22.0 |  |
| Endocrine; nutritional; and metabolic diseases and immunity disorders | 277 | 10.1 | 274 | 32.5 |  | 215 | 29.0 | 219 | 29.5 |  |
| Neoplasms | 75 | 2.7 | 8 | 0.9 |  | 10 | 1.3 | 8 | 1.1 |  |
| Infectious and parasitic diseases | 644 | 23.5 | 212 | 25.1 |  | 192 | 25.9 | 192 | 25.9 |  |
| All other CCS | 131 | 4.8 | 35 | 4.1 |  | 32 | 4.3 | 34 | 4.6 |  |
| **Hospital teaching status** | | | | | **<0.001** |  |  |  |  | **0.568** |
| Nonteaching | 2030 | 74.1 | 364 | 43.1 |  | 365 | 49.2 | 354 | 47.7 |  |
| Teaching | 709 | 25.9 | 480 | 56.9 |  | 377 | 50.8 | 388 | 52.3 |  |
| **Hospital size (number of beds)** | | | | | **<0.001** |  |  |  |  | **0.464** |
| ≤300 | 1066 | 38.9 | 145 | 17.2 |  | 133 | 17.9 | 144 | 19.4 |  |
| >300 | 1673 | 61.1 | 699 | 82.8 |  | 609 | 82.1 | 598 | 80.6 |  |
| **Geographic location (regions)** | | | | | **0.011** |  |  |  |  | **0.373** |
| Midwest | 313 | 11.4 | 122 | 14.5 |  | 84 | 11.3 | 101 | 13.6 |  |
| Northeast | 84 | 3.1 | 30 | 3.6 |  | 29 | 3.9 | 30 | 4.0 |  |
| South | 2107 | 76.9 | 642 | 76.1 |  | 589 | 79.4 | 562 | 75.7 |  |
| West | 235 | 8.6 | 50 | 5.9 |  | 40 | 5.4 | 49 | 6.6 |  |

^a^As determined by CCS software. ALaRMS, Acute Laboratory Risk of Mortality Score; C-NS, carbapenem nonsusceptible; C-S, carbapenem susceptible; CCS, Clinical Classification Software; ICU, intensive care unit; PDX, principal diagnosis.

**Supporting Table S2d.** Pre- and post-matching of baseline characteristics: Respiratory group 4 without bacterial pneumonia primary diagnosis and hospital onset

| **Variables** | **Pre-matching** | | | | | **Post-matching (1:1 match)** | | | | |
| --- | --- | --- | --- | --- | --- | --- | --- | --- | --- | --- |
|  | **C-S (n=2034)** | | **C-NS (n=489)** | |  | **C-S (n=463)** | | **C-NS (n=463)** | |  |
|  | n | Column % | n | Column % | ***P* value** | n | Column % | n | Column % | ***P* value** |
| **Sex** | | | | | **0.661** |  |  |  |  | **0.681** |
| Female | 690 | 33.9 | 171 | 35.0 |  | 168 | 36.3 | 162 | 35.0 |  |
| Male | 1344 | 66.1 | 318 | 65.0 |  | 295 | 63.7 | 301 | 65.0 |  |
| **Age group, years** | | | | | **<0.001** |  |  |  |  | **0.734** |
| 18-34 | 115 | 5.7 | 56 | 11.5 |  | 56 | 12.1 | 48 | 10.4 |  |
| 35-44 | 102 | 5.0 | 38 | 7.8 |  | 33 | 7.1 | 35 | 7.6 |  |
| 45-54 | 272 | 13.4 | 74 | 15.1 |  | 60 | 13.0 | 70 | 15.1 |  |
| 55-64 | 414 | 20.4 | 112 | 22.9 |  | 92 | 19.9 | 107 | 23.1 |  |
| 65-74 | 564 | 27.7 | 128 | 26.2 |  | 138 | 29.8 | 123 | 26.6 |  |
| 75-84 | 424 | 20.8 | 58 | 11.9 |  | 61 | 13.2 | 57 | 12.3 |  |
| 85 and over | 143 | 7.0 | 23 | 4.7 |  | 23 | 5.0 | 23 | 5.0 |  |
| **Payer** | | | | | **0.017** |  |  |  |  | **0.918** |
| Medicare | 1145 | 56.3 | 245 | 50.1 |  | 240 | 51.8 | 234 | 50.5 |  |
| Medicaid | 132 | 6.5 | 45 | 9.2 |  | 37 | 8.0 | 39 | 8.4 |  |
| Private/other | 757 | 37.2 | 199 | 40.7 |  | 186 | 40.2 | 190 | 41.0 |  |
| **Exposure risk (# of days from admission to onset of infection)** | | | | | **<0.001** |  |  |  |  | **0.653** |
| 1st quartile | 591 | 29.1 | 92 | 18.8 |  | 83 | 17.9 | 92 | 19.9 |  |
| 2nd quartile | 545 | 26.8 | 95 | 19.4 |  | 99 | 21.4 | 94 | 20.3 |  |
| 3rd quartile | 479 | 23.5 | 104 | 21.3 |  | 112 | 24.2 | 99 | 21.4 |  |
| 4th quartile | 419 | 20.6 | 198 | 40.5 |  | 169 | 36.5 | 178 | 38.4 |  |
| **ALaRMS Score** | | | | | **0.004** |  |  |  |  | **0.684** |
| 1st quartile | 560 | 27.5 | 120 | 24.5 |  | 128 | 27.6 | 115 | 24.8 |  |
| 2nd quartile | 518 | 25.5 | 101 | 20.7 |  | 103 | 22.2 | 98 | 21.2 |  |
| 3rd quartile | 488 | 24.0 | 121 | 24.7 |  | 104 | 22.5 | 112 | 24.2 |  |
| 4th quartile | 468 | 23.0 | 147 | 30.1 |  | 128 | 27.6 | 138 | 29.8 |  |
| **Number of hospital admissions in the 90 days before index admission** | | | | | **<0.001** |  |  |  |  | **0.659** |
| 0 | 1570 | 77.2 | 332 | 67.9 |  | 313 | 67.6 | 323 | 69.8 |  |
| 1 | 329 | 16.2 | 89 | 18.2 |  | 96 | 20.7 | 85 | 18.4 |  |
| >1 | 135 | 6.6 | 68 | 13.9 |  | 54 | 11.7 | 55 | 11.9 |  |
| **ICU admission status** | | | | | **0.920** |  |  |  |  | **0.893** |
| No | 812 | 39.9 | 194 | 39.7 |  | 181 | 39.1 | 183 | 39.5 |  |
| Yes | 1222 | 60.1 | 295 | 60.3 |  | 282 | 60.9 | 280 | 60.5 |  |
| **Mechanical ventilation status** | | | | | **<0.001** |  |  |  |  | **0.948** |
| No | 1384 | 68.0 | 232 | 47.4 |  | 225 | 48.6 | 224 | 48.4 |  |
| Yes | 650 | 32.0 | 257 | 52.6 |  | 238 | 51.4 | 239 | 51.6 |  |
| **Organism** | | | | | **<0.001** |  |  |  |  | **0.980** |
| *Pseudomonas aeruginosa* | 564 | 27.7 | 293 | 59.9 |  | 266 | 57.5 | 269 | 58.1 |  |
| Polymicrobial | 213 | 10.5 | 71 | 14.5 |  | 70 | 15.1 | 69 | 14.9 |  |
| Other gram-negative | 1257 | 61.8 | 125 | 25.6 |  | 127 | 27.4 | 125 | 27.0 |  |
| **PDX-based CCS disease category^a^** | | | | | **<0.001** |  |  |  |  | **0.991** |
| Missing PDX | 66 | 3.2 | 16 | 3.3 |  | 12 | 2.6 | 16 | 3.5 |  |
| Injury and poisoning | 317 | 15.6 | 100 | 20.4 |  | 94 | 20.3 | 91 | 19.7 |  |
| Diseases of the circulatory system | 496 | 24.4 | 49 | 10.0 |  | 54 | 11.7 | 49 | 10.6 |  |
| Diseases of the digestive system | 152 | 7.5 | 32 | 6.5 |  | 32 | 6.9 | 31 | 6.7 |  |
| Diseases of the respiratory system | 392 | 19.3 | 91 | 18.6 |  | 94 | 20.3 | 89 | 19.2 |  |
| Endocrine, nutritional, and metabolic diseases and immunity disorders | 50 | 2.5 | 31 | 6.3 |  | 27 | 5.8 | 29 | 6.3 |  |
| Neoplasms | 126 | 6.2 | 20 | 4.1 |  | 17 | 3.7 | 18 | 3.9 |  |
| Infectious and parasitic diseases | 270 | 13.3 | 111 | 22.7 |  | 93 | 20.1 | 102 | 22.0 |  |
| All other CCS | 165 | 8.1 | 39 | 8.0 |  | 40 | 8.6 | 38 | 8.2 |  |
| **Hospital teaching status** | | | | | **<0.001** |  |  |  |  | **0.740** |
| Nonteaching | 1543 | 75.9 | 266 | 54.4 |  | 269 | 58.1 | 264 | 57.0 |  |
| Teaching | 491 | 24.1 | 223 | 45.6 |  | 194 | 41.9 | 199 | 43.0 |  |
| **Hospital size (number of beds)** | | | | | **<0.001** |  |  |  |  | **0.312** |
| ≤300 | 696 | 34.2 | 75 | 15.3 |  | 64 | 13.8 | 75 | 16.2 |  |
| >300 | 1338 | 65.8 | 414 | 84.7 |  | 399 | 86.2 | 388 | 83.8 |  |
| **Geographic location (regions)** | | | | | **<0.001** |  |  |  |  | **0.575** |
| Midwest | 316 | 15.5 | 104 | 21.3 |  | 82 | 17.7 | 92 | 19.9 |  |
| Northeast | 41 | 2.0 | 19 | 3.9 |  | 18 | 3.9 | 17 | 3.7 |  |
| South | 1503 | 73.9 | 338 | 69.1 |  | 342 | 73.9 | 326 | 70.4 |  |
| West | 174 | 8.6 | 28 | 5.7 |  | 21 | 4.5 | 28 | 6.0 |  |

^a^As determined by CCS software. ALaRMS, Acute Laboratory Risk of Mortality Score; C-NS, carbapenem nonsusceptible; C-S, carbapenem susceptible; CCS, Clinical Classification Software; ICU, intensive care unit; PDX, principal diagnosis.
